# Effects of Diet Quality and Temperature on Stable Fly (Diptera: Muscidae) Development

**DOI:** 10.3390/insects10070207

**Published:** 2019-07-16

**Authors:** Melina Florez-Cuadros, Dennis Berkebile, Gary Brewer, David B. Taylor

**Affiliations:** 1 Department of Entomology, University of Nebraska – Lincoln, Lincoln, NE 68583-0816, USA; 2 Agroecosystems Management Research Unit, USDA-ARS, Lincoln, NE 68583-0937, USA

**Keywords:** *Stomoxys calcitrans*, Life history, Survival, Fitness

## Abstract

The effects of diet quality and temperature on the development time and size of stable flies, *Stomoxys calcitrans* (L.), was evaluated. Both development time and size varied relative to diet quality and temperature, and their effects were additive. Diet quality and temperature made similar contributions to the variance in size whereas temperature was responsible for >97% of the variance in development time. Regression analysis predicted the shortest development time, egg to adult, to be 12.7 days at 32 °C and 70% nutrients. Egg to adult development varied curvilinearly relative to diet quality and temperature on the degree day 10 (DD_10_) scale taking 261 DD_10_ at 30 °C and 50% nutrients. The thermal threshold was 11.5 °C with a thermal constant of 248. Very few stable flies developed to adult on the poorest diet (12.5% nutrients) and adults emerged from fewer than 1% of the puparia at 35 °C. The heaviest pupae (15.4 mg) were produced with the 100% diet at 15 °C and adults had a higher probability of emerging successfully from heavier puparia. The length of the discal-medial cell of adult wings had a cubic relationship with puparia weight and peaked at 21 °C. Egg to pupariation survival was predicted to peak at 27 °C and 71% diet whereas puparia to adult survival peaked at 24 °C and 100% diet. Diet quality and temperature had no effect on sex ratio and the rate of development did not differ between the sexes. Female stable flies were ≈5% larger than males. Composite metrics for egg to pupariation and egg to adult fitness were developed. The optimum for puparia fitness was 29 °C and 78% diet quality and for adult fitness 25 °C and 83% diet quality. Diet accounted for 31% of the variance in pupal fitness and 24% of the variance in adult fitness whereas temperature accounted for 17% and 20%, respectively.

## 1. Introduction

Fecundity and development rate are two key elements for characterizing the life history of an organism [1]. Body size is a primary determinant of fecundity and fitness in insects [2,3,4] and in holometabolous insects, body size and development rate are largely determined by environmental parameters experienced during the larval stage [5]. Primary among those environmental parameters are resources (food) and temperature. The responses of insects to temperature and resource limitations differ among, even closely related species [1]. Most insects increase their development rate and decrease in size relative to increasing temperature [6] and reduce development rate and size with limited resources. However, some species adjust by increasing development time and metamorphose at a size similar to their unrestricted counterparts while others retain the normal development period and metamorphose at a smaller size. These differences are partially dependent upon the stability of the larval habitat and time-dependent mortality within it [7].

Stable flies, *Stomoxys calcitrans* (L.) (Diptera: Muscidae), are pests of livestock, wildlife, and humans throughout much of the world. Male and female stable flies require bloodmeals for reproductive success [8]. Their painful bites reduce productivity of livestock, molest wildlife, and disrupt recreational activities of humans. Although most of the damage caused by this pest is a direct product of their bloodfeeding activity, in some regions of the world, they are important vectors of livestock and wildlife pathogens [9].

The relationship between temperature and development rate of stable flies has been evaluated in several studies [10,11,12,13,14] and the development rates of those studies have been used to evaluate stable fly population dynamics [15,16,17]. However, none of these studies include effects of diet quality on developmental rate or size. In field trapping studies, we have observed greater than two-fold variation in the size of adult stable flies (Figure S1). Smaller flies are especially abundant in mid-summer and late fall—seasons when stable fly populations are ebbing. 

Stable fly larvae develop in a broad variety of decomposing vegetative materials [18] and presumably the quality of those substrates relative to stable fly nutritional and environmental needs vary as well. Stable fly developmental substrates are ephemeral and their suitability for stable fly development is temporally dynamic [19,20,21]. The objective of this study was to evaluate the effects of diet quality on the development of immature stable flies and the interactions of diet quality with temperature.

## 2. Materials and Methods 

Stable flies were derived wild flies collected in 2004 near Firth, Nebraska and maintained using the methods outlined in Friesen et al. [22]. The experimental design was a complete block with four diets and 5 temperatures. For each treatment, three replicate 148 mL (5 oz) cups were prepared with 100 g of diet and 0.025 mL of stable fly eggs (≈185 eggs). Diets were prepared in bulk and allocated to cups. Stable fly eggs were placed in a depression in the diet and covered lightly. Cups were covered with cloth and randomly allocated to environmental chambers maintained at 15, 20, 25, 30, and 35 °C. Diets were based upon a standard diet referred to as the 100% diet (Table 1). Nutrients were reduced in the other diets by replacing nutrient ingredients (wheat bran and fish meal) with the inert bulking ingredient (vermiculite) to result in diets with 50%, 25%, and 12.5% of the nutrient ingredients of the 100% diet. Tap water was added to each diet to yield a final concentration of 68% water, by weight. The entire study was repeated four times.

Cups were inspected daily and puparia were removed, weighed (Acculab, LA-110 Balance, Goettingen, Germany), and placed individually in wells of 48-well plates. The date of pupariation and puparia weight were recorded. Plates were returned to their respective environmental chamber and inspected daily for adult emergence. Cups were discarded when no live stable fly larvae were observed during inspection. Once adult emergence was complete (no new adults emerging for 2 weeks), adults were sexed and both wings were mounted on glass slides with clear fingernail polish. Length of the discal medial (D-M) cell of each wing was measured at 50× (Dino-lite Edge 5MP digital microscope, Torrance, CA, USA). For each fly that completed development to the adult stage, egging date, pupariation date, emergence date, sex, and length of D-M cell of right and left wing were recorded. The mean of the two wing measurements was used for all analyses except for asymmetry. For those flies that pupariated but failed to successfully emerge as adults, we lack emergence date, sex and D-M cell length data.

Statistical analysis. Development time, egg to pupariation and pupariation to adult emergence, were analyzed with general linear mixed models (GLMM, Proc GLIMMIX, SAS 9.4, Cary, NC, USA). Diet and temperature were considered continuous variables to develop regression models to describe changes in developmental parameters relative to diet and temperature. Squared terms for diet and temperature were included to determine if the responses were linear or curvilinear. Non-significant terms were removed from models in a stepwise fashion (α = 0.05 for all analyses). Threshold and thermal constant values were calculated with the method of Arnold [23]. In addition to the individual parameters, composite measures of the effects of diet and temperature, fitness, were determined for each cup. For puparia, fitness was calculated as the (number of puparia × mean puparia weight)/mean development time (d). Similarly, adult fitness was calculated as (number of adults × mean length of D-M cell)/mean development time (d). A symmetry index (SI) was calculated from the right and left wing D-M cell measurements as 1 − |(Right − Left)/(Right + Left)|. SI varied from 0 to 1 with a perfect match between sides being 1. The SI was evaluated relative to diet, temperature, and fitness with the beta distribution.

## 3. Results 

### 3.1. Development Time

**Figure 1:**
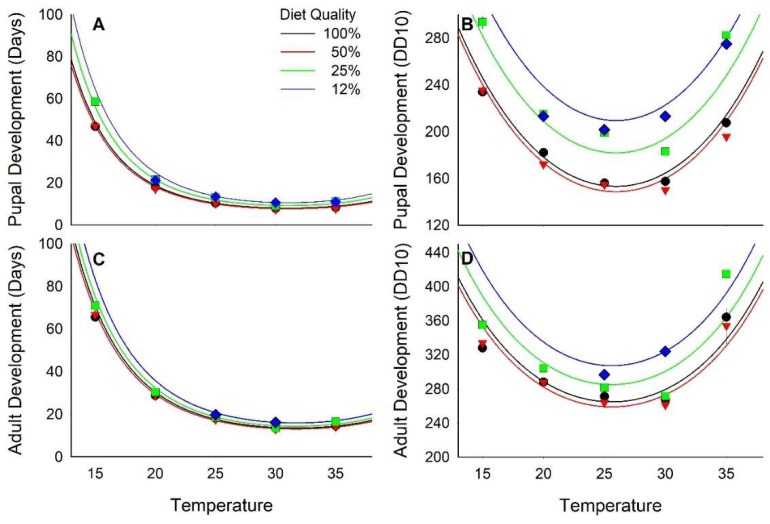
Effect of diet quality and temperature on development time. (**A**) and (**B**) are time to pupariation; (**C**) and (**D**) are time to adult emergence. (**A**) and (**C**) are in units of days; (**B**) and (**D**) are in units of Degree Days_10_. Lines are regression equations and bars are standard errors.

Development time to pupariation ranged from nearly 60 days (d) at 15 °C to <8 d at 30 °C, decreasing curvilinearly relative to both diet quality and temperature (Figure 1A and Figure S2; Table 2 and Table 3). On a degree day scale with a 10 °C threshold (DD_10_), development to pupariation ranged from 150 DD_10_ when reared at 25 and 30 °C on diets with ≥50% nutrients to nearly 300 DD_10_ at 15 and 35 °C on diets with ≤25% nutrients (Figure 1B and Figure S2; Table 2 and Table 3). The regression model indicated that minimum pupariation time was 139 DD_10_ at 26°C and with a 73% nutrient diet. 

The development from egg to adult emergence also varied relative to diet and temperature. No adults emerged from treatments with 12% diet at 15, 20, and 35 °C. The relationship of development to adult emergence relative to diet and temperature was curvilinear (Figure 1C; Table 2 and Table 3) with the fastest development, 13 d, at 30 °C and 50% diet nutrients. Development was delayed to >70 d at 15 °C and ≤25% nutrients. Temperature accounted for 97% of the variance in development time whereas diet accounted for only 0.3% of the variance. On the DD_10_ scale, development to adult emergence ranged from ≈261 DD_10_ when reared at 30 °C on diets with ≥50% nutrients to >400 DD_10_ when reared at 35 °C on diets with ≤25% nutrients (Figure 1D, Table 2 and Table 3). Mean development time for the 50 and 100% diets across all temperatures was 275.3 ± 26.1 DD_10_. The effects of diet quality and temperature on development time in DD_10_ were additive.

Diet had a significant effect on the intercept of the regression of development rate (1/development time [d]) relative to temperature (F_2,118_ = 15.16, *p* < 0.01; 12% diet treatment excluded because adults were recovered at only two temperatures) but no effect on the slope (F_2,116_ = 0.06, *p* = 0.94). Thus, the developmental constant, 244 DD, did not vary relative to diet, but the thermal threshold was higher when reared on 25% nutrient diet, 12.43 °C (t_118_ = −4.43, *p* < 0.01), than when reared on either 50% or 100% diets, 11.33 and 11.51 respectively, which did not differ from each other (t_118_ = 0.98, *p* = 0.33). Overall, for the 25%–100% diets pooled, the thermal threshold was 11.5 and the thermal constant was 248.

### 3.2. Weight and Size

**Figure 2:**
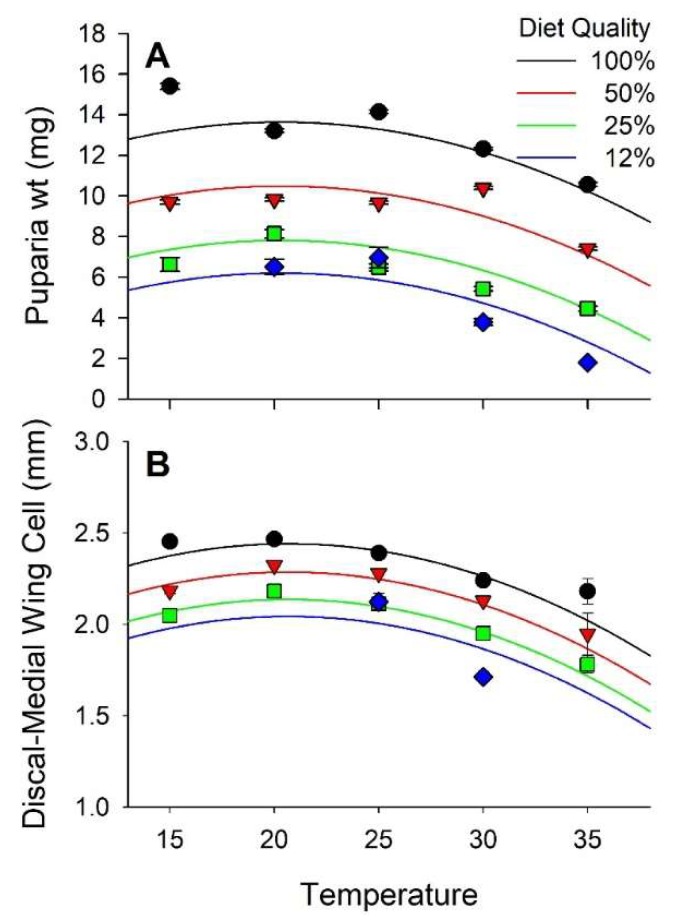
Effect of diet quality and temperature on size of stable flies. (**A**) is weight of the puparia; (**B**) is length of the discal medial cell of the wing.

Stable fly puparia ranged in weight from <4 mg when reared at 30 and 35 °C on the 12% nutrient diet to >15 mg when reared at 15 °C on the 100% nutrient diet. Diet and temperature both had significant effects on the weight of puparia, but no interaction between diet and temperature was observed. Weight increased curvilinearly in relation to diet nutrients and varied curvilinearly with respect to temperature peaking at 20.3 °C (Figure 2A and Figure S2, Table 2 and Table 3). Diet accounted for 43% of the observed variance in puparia weight whereas temperature accounted for only 8%.

**Figure 3:**
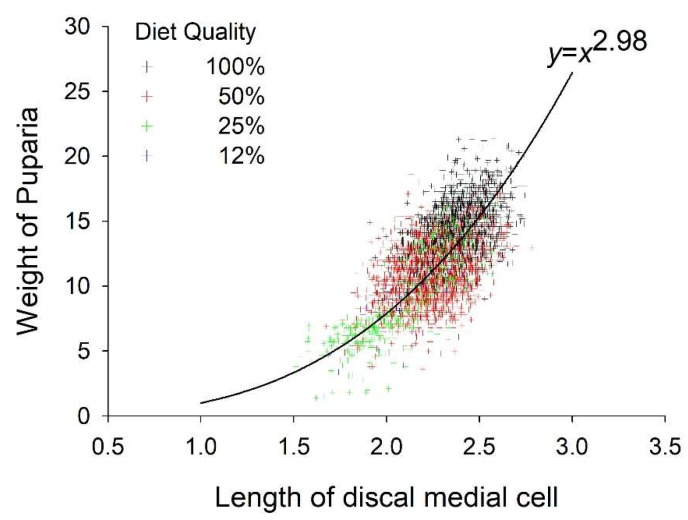
Relationship between weight of the puparia and length of the discal medial cell. Points are the observed data and the line is the exponential model.

Puparia weight and length of the D-M cell were positively correlated (r = 0.66, *p* < 0.01). Weight of the puparia increased exponentially relative to the length of the D-M cell (Figure 3). The D-M cell of the wing ranged in length from 1.7 mm when reared at 30°C on 12% diet to >2.4 mm when reared at 15 and 20 °C on 100% diet. Diet and temperature both had significant effects on the length of the D-M cell whereas their interaction did not. D-M cell length increased curvilinearly relative to diet throughout the experimental range and peaked at 20.7 °C (Figure 2B, Table 2 and Table 3). Diet accounted for 22% of the variance in D-M cell length whereas temperature accounted for 19%. 

The SI increased as diet quality increased (t_122_ = 4.27, *p* < 0.01) and as temperature declined (t_122_ = −7.04, *p* < 0.01). The final model was logit (SI) = 5.007 + 0.225D − 0.024T. Mean SI per cup was not correlated with puparia (r = 0.044, *p* = 0.62) or adult (r = 0.038, *p* = 0.66) fitness. Individual SI values were weakly correlated with puparia weight (r = 0.053, *p* < 0.01) and mean D-M cell length (r = 0.098, *p* < 0.01).

### 3.3. Survival

**Figure 4:**
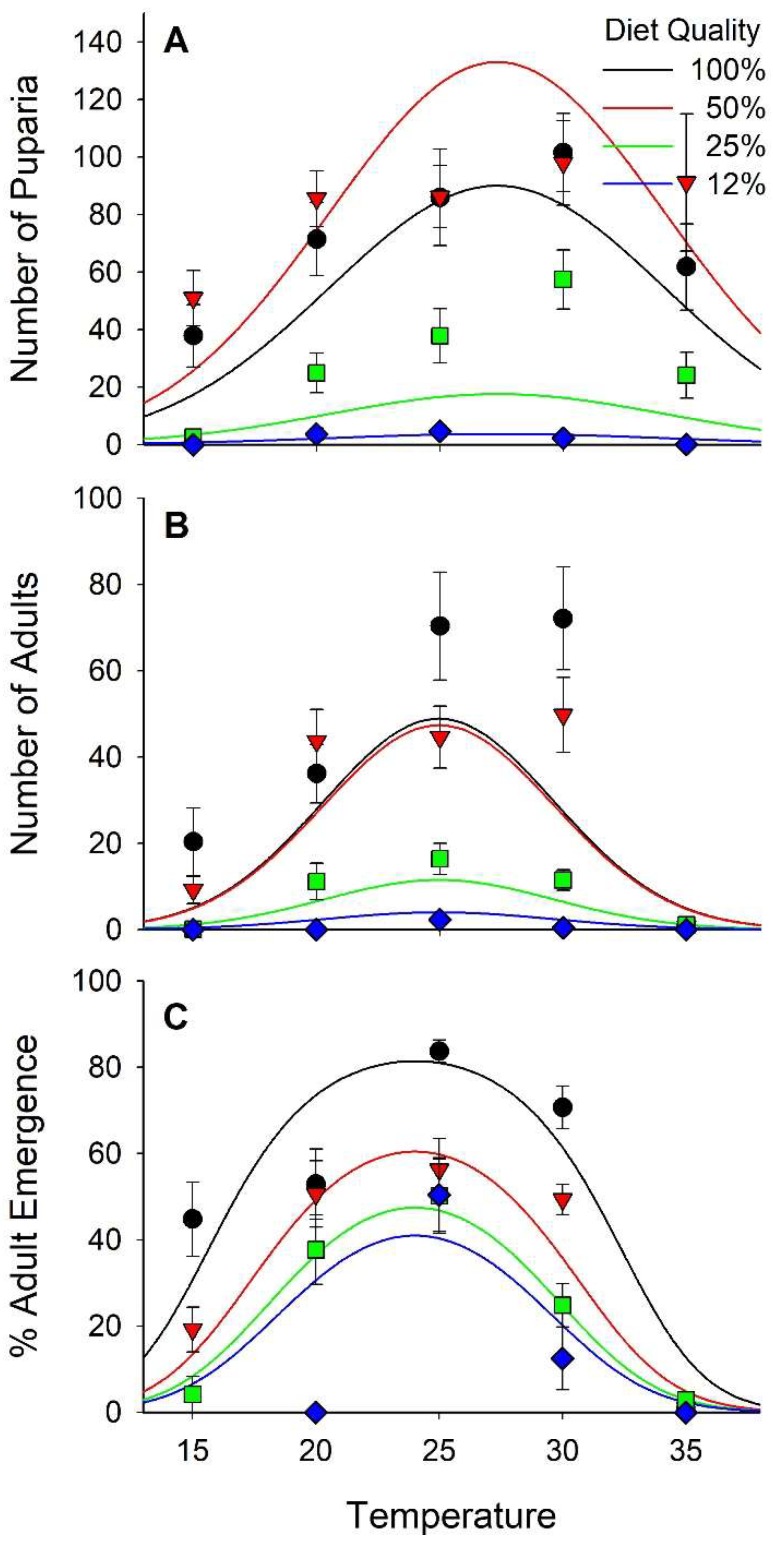
Number of puparia (**A**) and number of adults (**B**) per cup and percentage of the puparia from which adults successfully emerged (**C**). Each cup received ≈185 stable fly eggs.

**Figure 5:**
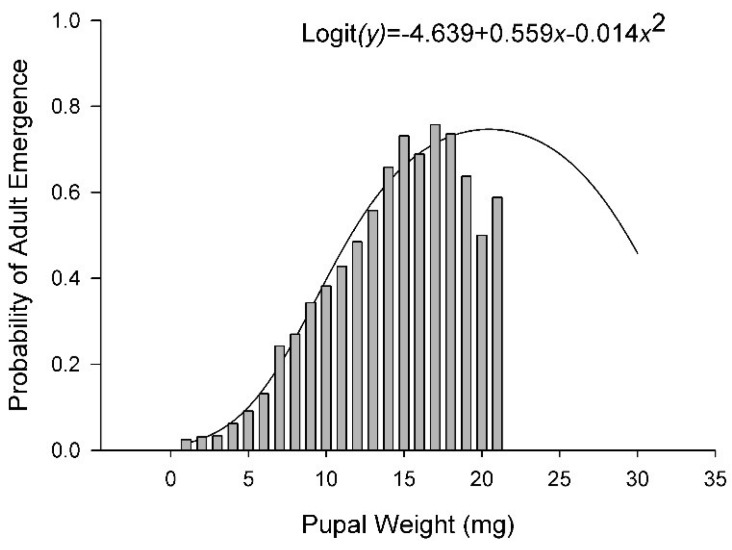
Probability of successful adult emergence relative to pupal weight. Solid line represents the logistic model. Bars represent observed data. Data are from all temperatures and diets combined.

The number of eggs delivered per cup with the calibrated pipette was evaluated with 10 replicate samples. A mean of 185 (SD = 13.1) eggs were delivered by the pipette to each cup. In total, 10,606 puparia and 4676 adults were recovered in this study. The number of puparia per cup ranged from 0 at 15 °C on 12% diet to 102 at 30 °C on 100% diet. The number of puparia varied curvilinearly relative to both diet and temperature with peaks at 27.3 °C and 71% diet nutrients (Figure 4A and Figure S2, Table 2 and Table 3). Diet accounted for 42% of the variance in the number of puparia per cup and temperature accounted for 8%. The number of adults ranged from 0 at 15, 20, and 35 °C on the 12% diet to >70 at 25 and 30 °C on 100% diet. Overall, the number of adults per cup varied curvilinearly relative to both diet nutrients and temperature, peaking at 77% nutrients and 24.7 °C (Figure 4B, Table 2 and Table 3). Adults emerged from only 1% of the puparia maintained at 35 °C whereas >60% of the puparia produced adults at 25 °C (Figure 4C). Diet accounted for 14% of the variance in successful emergence of an adult from a puparia whereas temperature account for 41%. The probability of an adult successfully emerging from a puparia varied curvilinearly relative to the weight of the puparia peaking at 17–18 mg (Figure 5). The regression model derived from our data predicted peak probability of successful emergence to be at 20 mg, however, very few pupae in that size range were recovered and those that were had lower emergence success than puparia weighing 17–18 mg.

### 3.4. Sex

**Figure 6:**
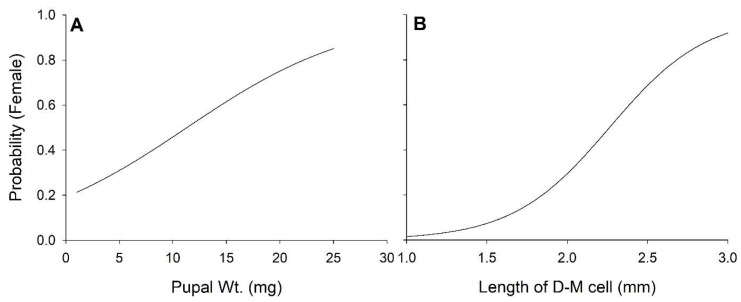
Relationship between size, pupal weight (**A**) and length of discal-medial wing cell (**B**), and sex.

Overall, a slight excess of females, 1.08:1 (χ^2^_1_ = 6.95, *p* < 0.01), was observed among the emerged adults. The probability of a fly being male or female was independent of diet (F_3,4441_ = 0.92, *p* = 0.43), temperature (F_4,4441_ = 2.03, *p* = 0.09), and their interaction (F_9,4441_ = 1.33, *p* = 0.22). Development time from egg to pupariation (F_1,4438_ = 0.23, *p* = 0.11) and egg to adult emergence (F_1,4437_ = 0.42, *p* = 0.52) did not differ between the sexes. Puparia from the female stable flies that emerged were on average 0.65 mg (5.4%) heavier than those from male flies that emerged (F_1,4399_ = 75.28, *p* < 0.01) and the discal-medial wing cell of females was on average 0.1 mm (4.8%) longer than that of males (F_1,4250_ = 313.40, *p* < 0.01; Figure 6).

### 3.5. Fitness

**Figure 7:**
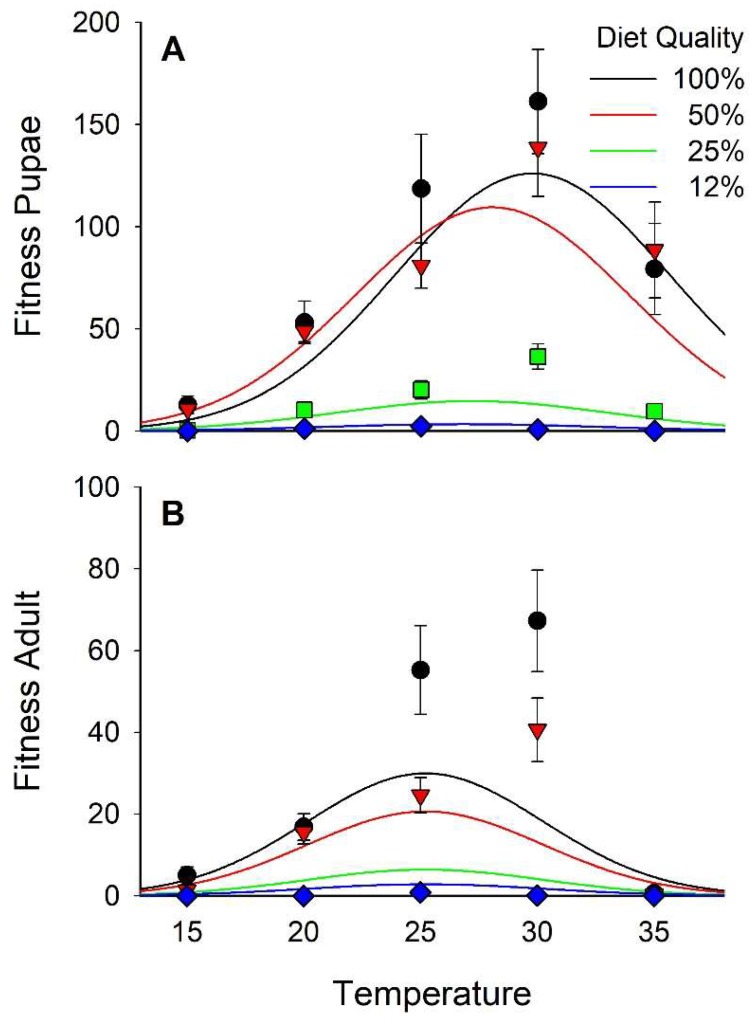
Fitness of pupae (=[Number of pupae per cup × Mean pupal weight]/Mean development time [d]; $\bar{x}$ ± SEM; **A**) and adults (=[Number of adults per cup × mean length of discal medial cell (mm)]/Mean development time [d]; **B**).

High levels of mortality were observed under some of our experimental conditions, especially in puparia, resulting in nearly 2-times more records for puparia than for adults. In addition, no adults were recovered from some of the diet × temperature treatments. Therefore, we analyzed fitness for egg to pupariation and for egg to adult emergence. Pupal and adult fitness varied in a curvilinear manner relative to both diet and temperature (Figure 7 and Figure S2; Table 2 and Table 3). The optima for pupal fitness was 29°C with 78% diet. For adults, the optimum was 25°C with 83% diet. Diet accounted for 31% of the variance in pupal fitness and 24% of the variance in adult fitness, whereas temperature accounted for 17% and 20%, respectively.

## 4. Discussion

### 4.1. Development Time and Size

Stable flies reared as larvae under diet stress conditions developed at a reduced rate for a period approximately equal to that of their peers developing with adequate nutrients resulting in small adults. This response is similar to that observed in *Sepsis cynipsea* (L.) (Diptera: Sepsidae) [1]. Development rate is primarily dependent upon temperature whereas size is primarily dependent upon diet quality. Overall, development rates are similar to those reported by Lysyk [13,24] although consistently slightly longer. Median development time at 25 °C on the 100% diet was ≈0.7 d longer than that reported by Lysyk [13]. The fastest development was observed between 29 and 31 °C. However, pupal weight and D-M wing cell were largest at 20 and 21 °C, respectively.

Based upon the dynamics of a wild population, Lysyk [24] determined the average stable fly generation time to be ≈258 DD_10_. Based upon the 50% and 100% diets between 20 and 30 °C, we observed a mean immature development time of 275 DD_10_. The DD_10_ values were relatively consistent between 20 and 30 °C. Above and below those temperatures, physiological development rates decreased significantly. The thermal threshold observed in this study, 11.4 °C, is similar to the 12.3 °C observed by Larsen and Thomsen [10]. This verifies using DD_10_ for evaluating stable fly population dynamics [24] following arguments for standardization of threshold temperatures [25].

The size of holometabolous insects is dependent upon the conditions experienced by the larvae [5]. Therefore, adult size can be used to evaluate parameters of developmental habitats. For stable flies, this is especially important because flies can migrate long distances [26,27] and develop in habitats distinct from where they are being pestiferous [16]. The ability to associate adult flies with their developmental sites would assist management efforts.

Although several metrics can be used to evaluate the size of flies including, larva, puparia, and adult weight, wing length, both total and partial, and head capsule width among others, we opt to use the length of the D-M cell of the wing. Wings can be recovered from adult flies collected by many methods, mounted on slides easily, and stored without shrinkage or collapse for later analysis and voucher. The D-M cell is the longest cell of the stable fly wing, thus the easiest to measure accurately. Frequently, the wings of field collected stable flies are damaged making measurement of total length difficult. However, the D-M cell, being in the middle of the wing, is usually intact. The length of the D-M cell is correlated with pupal weight, and therefore a valid metric for evaluating size. The relationship, as expected based on geometry, is cubic. 

Stressing factors, including limited nutrition and temperature, can lead to developmental instability which may be reflected by asymmetry of bilateral structures or fluctuating asymmetry [28,29]. Symmetry of the length of the D-M wing cell increased relative to the quality of the diet and decreased relative to temperature indicating that both high temperature and poor diet quality increase developmental stress. However, the correlations between SI and size, a commonly used metric for quality, were quite weak (r < 0.1) indicating that SI has little value for retrospectively assessing developmental substrate quality or conditions for individual stable flies.

The primary purpose of developing life history models is to relate the results to observations of population dynamics in the field. Part of this is an evaluation of how the developmental environment in the laboratory relates to that in the field. The flies emerging from the different diets in this experiment were smaller than wild flies collected in the field. Additional studies are needed on diet quality to understand why stable flies developing on our laboratory diet are smaller on average than wild flies collected on sticky traps in the field and how this difference may be affecting the applicability of results of laboratory experiments relative to field conditions. The 100% diet flies were smaller than the 15th percentile of wild flies (Figure S1).

### 4.2. Survival

Survival, both egg to pupariation and pupariation to adult emergence, varied with diet quality and temperature. As with previous reports, very few stable flies survived to adult emergence when developing at 35 °C. Most of the mortality was observed in puparia. Larsen and Thomsen [10] noted that mortality was not elevated when young pupae reared at lower temperatures were moved to 35 °C indicating the critical stage was in the larvae or larva to pupa transition. Likewise, very few adults emerged from the 12% diet treatments. The number of puparia was similar between the 50% and 100% diet treatments, but adult emergence was much higher in the 100% treatment. Larger pupae have a higher probability of successfully producing an adult fly than do smaller pupae.

### 4.3. Sex

The overall sex ratio of the flies recovered in this study, 1.08 ♀♀/♂ was similar to that observed in flies emerging from natural developmental sites in the field, 1.06 ♀♀/♂ [20]. Diet quality and temperature have no effect on sex ratio, and development rates for male and female stable flies are similar. Female stable flies are ≈5% larger than males, but the size dimorphism is not diagnostic. 

### 4.4. Fitness

Diet and temperature vary in their effects on different aspects of stable fly development. Higher temperatures promote rapid development while lower temperatures promote larger size. We used two metrics to evaluate how size, survival, and rate of development combine to contribute to the overall fitness of the flies. The first was mg of puparia/development day/experimental unit (cup) and the second mm of D-M cell length/development day/experimental unit. The contributions of diet and temperature to adult fitness were about equal whereas diet had a larger contribution to puparia fitness. Interestingly, fitness was not optimal on our standard, 100%, diet. 

The models indicated the optimal diet was ≈80% of standard nutrients. Optimal temperature for puparia fitness was 29 °C and for adult fitness 25 °C. Larsen and Thomsen [10] observed optimal growth at 30–33 °C, but they did not take size or survival into consideration. Adult emergence remained high at 30 °C, but dropped dramatically to less than 2% at 35 °C. The mathematical models may fail to reflect the steep slope of the lethal effects of temperature at some point between 30 and 35 °C. Further studies are needed to refine the lethal temperature and to determine the sensitive stage and age.

## 5. Conclusions

The development rate of stable flies is determined primarily by temperature whereas size is dependent upon both the quality of the developmental substrate and temperature. Egg to adult development was completed in less than 13 days at 30 °C but required over 70 days at 15 °C. The thermal threshold for stable flies was 11.4°C and the thermal constant 247. Egg to adult development required ≈275 DD_10_. Puparia of stable flies developing at low temperatures and on high quality substrate weighed more than 15 mg while those of flies developing at higher temperatures and on poor quality substrate weighed less than 4 mg. Most of the flies reared at 35°C died during the post-larval preimaginal stage. Male and female stable flies develop to adulthood at the same rate even though females are ≈5% larger. Based upon regression models, optimal temperature for stable fly fitness is 25 °C. Because stable flies developing in natural habitats also experience variation in diet quality and temperatures our results provide an explanation for seasonal variation in fly size. Our results also suggest optimal diet quality and temperature conditions for the laboratory rearing of stable flies. 

## Figures and Tables

**Table 1:** Diets were based upon standard laboratory diet (Friesen et al. 2018 [22]). Percentages are by weight. Dry ingredients were mixed with tap water to yield 68% water.

Ingredient (%)			
% Nutrients	Wheat Bran	Fish Meal	Vermiculite
100	59	13	28
50	30	8	63
25	15	3	82
12	7	2	91

**Table 2:** Statistics for GLMM models with diet and temperature considered continuous variables.

	Transformation	df	Diet		Diet ^2^		Temp		Temp ^2^		Diet × Temp	
			*t*	*p*	*t*	*p*	*t*	*p*	*t*	*p*	*t*	*p*
Pupal Dev. (d)	Logn	161	−7.18	<0.01	6.35	<0.01	−30.38	<0.01	23.55	<0.01	−1.13	0.26
Pupal Dev. (DD_10_)	Square Root	161	−7.72	<0.01	6.68	<0.01	−14.56	<0.01	14.24	<0.01	−0.86	0.39
Adult Dev. (d)	Logn	135	−5.07	<0.01	4.79	<0.01	−29.95	<0.01	22.07	<0.01	−0.68	0.50
Adult Dev. (DD_10_)	Logn	135	−5.11	<0.01	4.59	<0.01	−12.14	<0.01	11.69	<0.01	−0.10	0.92
Pupal wt.		161	4.86	<0.01	−2.39	0.02	2.86	<0.01	−3.57	<0.01	−1.31	0.19
Adult D−M cell		130	4.12	<0.01	−2.31	<0.01	4.95	<0.01	−5.78	<0.01	−1.07	0.29
No. Pupae	Logn (N+1)	71	11.18	<0.01	−9.24	<0.01	4.81	<0.01	−4.38	<0.01	0.28	0.78
No. Adults	Logn (N+1)	71	6.77	<0.01	−5.20	<0.01	7.96	<0.01	−8.02	<0.01	−1.26	0.21
% Adult Emer.	Logit	58	7.74	<0.01	−1.87	0.07	10.03	<0.01	−10.25	<0.01	−1.24	0.22
Fitness Puparia	Logn (N+1)	70	8.01	<0.01	−8.92	<0.01	6.21	<0.01	−5.94	<0.01	2.17	0.03
Fitness Adult	Logn (N+1)	71	5.59	<0.01	−4.02	<0.01	8.12	<0.01	−8.01	<0.01	0.17	0.86

**Table 3:** Parameters for regression models describing stable fly developmental parameters relative to diet and temperature.

Parameter	Intercept	Diet	Diet ^2^	Temp	Temp ^2^	Diet × Temp
	*b* ± SEM	*b* ± SEM	*b* ± SEM	*b* ± SEM	*b* ± SEM	*b* ± SEM
Pupal Dev. (d)	9.33 ± 0.18	−1.489 ± 0.207	1.033 ± 0.163	−0.439 ± 0.014	0.007 ± 0.0003	
Pupal Dev. (DD_10_)	34.55 ± 1.30	−10.65 ± 1.38	7.28 ± 1.09	−1.46 ± 0.10	0.028 ± 0.002	
Adult Dev. (d)	8.84 ± 0.16	−0.947 ± 0.187	0.677 ± 0.141	−0.377 ± 0.013	0.0059 ± 0.0003	
Adult Dev. (DD_10_)	7.68 ± 0.16	−0.822 ± 0.161	0.572 ± 0.125	−0.146 ± 0.012	0.0028 ± 0.0002	
Pupal wt.	−2.09 ± 2.92	15.09 ± 3.10	−5.86 ± 2.45	0.639 ± 0.224	−0.0157 ± 0.0044	
Adult D−M cell	1.07 ± 0.22	0.884 ± 0.215	−0.383 ± 0.165	0.084 ± 0.017	−0.002 ± 0.0004	
No. Pupae	−8.75 ± 1.47	17.31 ± 1.55	−12.12 ± 1.31	0.589 ± 0.122	−0.011 ± 0.0025	
No. Adults	−13.08 ± 1.59	11.35 ± 1.68	−7.39 ± 1.42	1.06 ± 0.13	−0.021 ± 0.0027	
% Adult Emer.	−16.86 ± 1.64	2.10 ± 0.27		1.35 ± 0.13	−0.028 ± 0.0027	
Fitness Pupal	−10.08 ± 1.42	13.84 ± 1.73	−10.73 ± 1.20	0.706 ± 0.114	−0.013 ± 0.0023	0.087 ± 0.040
Fitness Adult	−12.42 ± 1.46	8.61 ± 1.54	−5.24 ± 1.31	0.988 ± 0.122	−0.020 ± 0.0025	

## Supplementary Materials

The following are available online at https://www.mdpi.com/2075-4450/10/7/207/s1, Figure S1: Empirical distribution of D-M cell length of experimental stable flies relative to that of wild flies, Figure S2: Contour map of development time, size and fitness relative to diet quality and temperature.
