# Supplementary material for: Effects of Diet Quality and Temperature on Stable Fly (Diptera: Muscidae) Development
Source: Insects. 2019 Jul 16;10(7):207. doi: 10.3390/insects10070207 (PMC6681194; doi:10.3390/insects10070207)
Supplement: Supplementary file 1 [file insects-10-00207-s001.pdf]

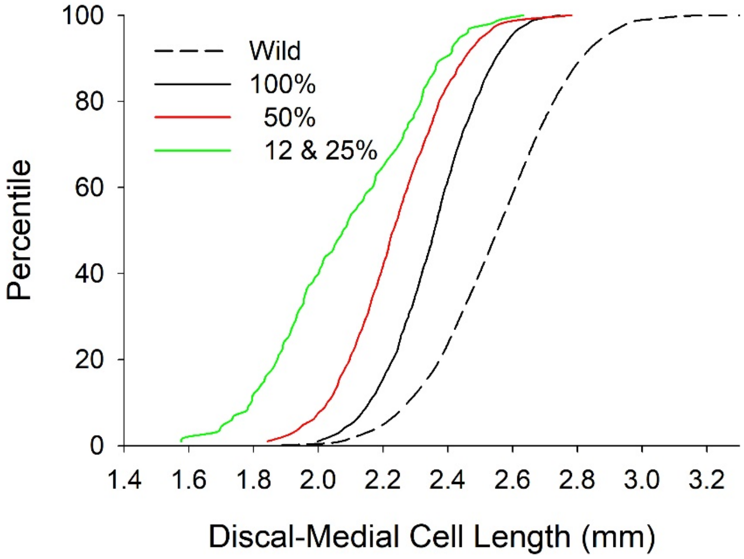

Figure S1. Empirical distribution of D-M cell length of >3,500 stable flies collected on Alsynite sticky traps in eastern Nebraska (Taylor et al. 2017) compared with distributions of flies from 100, 50, and pooled 25 and 12% diets in this study.

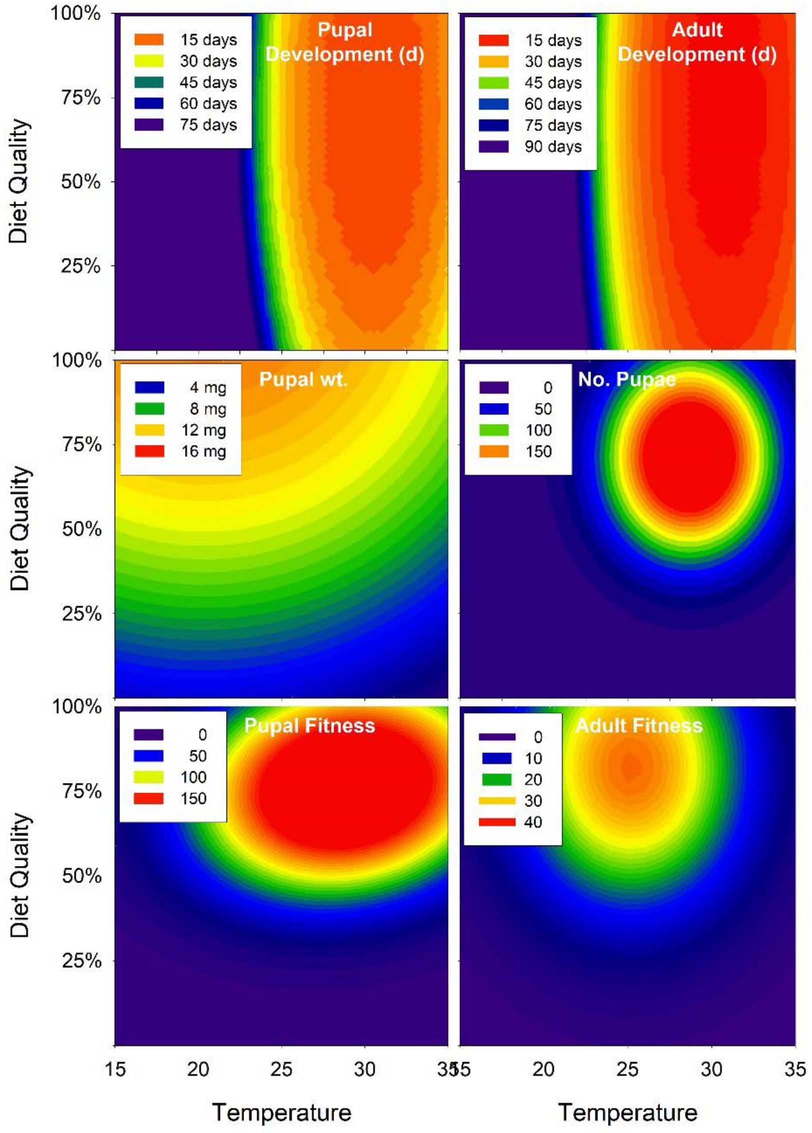

Figure S2. Contour diagrams of development time, to pupariation and to adult emergence, pupal weight, number of pupae per cup, and fitness, pupal and adult, relative to diet quality and temperature.
